# Carotenoid Biosynthesis: Genome-Wide Profiling, Pathway Identification in *Rhodotorula glutinis* X-20, and High-Level Production

**DOI:** 10.3389/fnut.2022.918240

**Published:** 2022-06-17

**Authors:** Shaobo Bo, Xiaoxia Ni, Jintang Guo, Zhengyang Liu, Xiaoya Wang, Yue Sheng, Genlin Zhang, Jinfeng Yang

**Affiliations:** Key Laboratory for Green Processing of Chemical Engineering of Xinjiang Bingtuan, School of Chemistry and Chemical Engineering, Shihezi University, Shihezi, China

**Keywords:** genomic analysis, comparative genomes, *R. glutinis*, carotenoid pathway, heterologous expression

## Abstract

*Rhodotorula glutinis*, as a member of the family *Sporidiobolaceae*, is of great value in the field of biotechnology. However, the evolutionary relationship of *R. glutinis* X-20 with *Rhodosporidiobolus*, *Sporobolomyces*, and *Rhodotorula* are not well understood, and its metabolic pathways such as carotenoid biosynthesis are not well resolved. Here, genome sequencing and comparative genome techniques were employed to improve the understanding of *R. glutinis* X-20. Phytoene desaturase (crtI) and 15-cis-phytoene synthase/lycopene beta-cyclase (crtYB), key enzymes in carotenoid pathway from *R. glutinis* X-20 were more efficiently expressed in *S. cerevisiae* INVSc1 than in *S. cerevisiae* CEN.PK2-1C. High yielding engineered strains were obtained by using synthetic biology technology constructing carotenoid pathway in *S. cerevisiae* and optimizing the precursor supply after fed-batch fermentation with palmitic acid supplementation. Genome sequencing analysis and metabolite identification has enhanced the understanding of evolutionary relationships and metabolic pathways in *R. glutinis* X-20, while heterologous construction of carotenoid pathway has facilitated its industrial application.

## Graphical Abstract

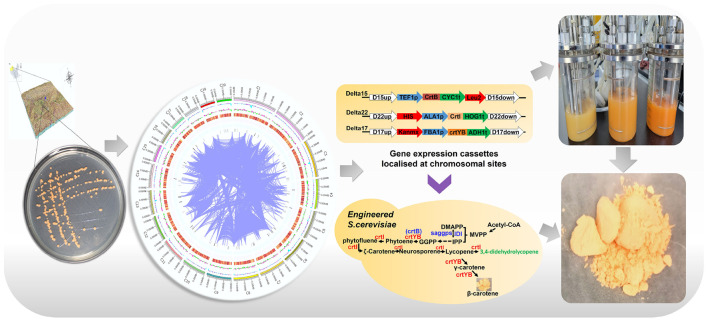

## Highlights

- High quality of genome map of *R. glutinis* X-20 was assembled.
- Carotenoid pathway and new enzymes were identified by comparative genome.
- High level of carotenoids was produced in *S. cerevisiae* expressing crtI and crtYB.

## Introduction

Wet oleaginous yeast has been widely studied because of its great application value in bioenergy (1). *Rhodotorula glutins* is a kind of typical wet oleaginous yeast, which is widely found in the ecological environment. Most of them live in the soil and the sea, but a small number grow by adhering to plants or symbiosing with other organisms (2). *Rhodotorula glutinis* can convert low-cost carbohydrates into lipids (3). The proportion of lipids in cells dry mass can reach 70%, mainly including monounsaturated fatty acids (C18:1), saturated fatty acids (C16:0) and polyunsaturated fatty acids (C18:2) (4, 5). Carotenoids are another important product from *R. glutinis*. For example, *R. glutinis* from the beer wastewater with high concentration of sugar (24.34 g⋅L^–1^ maltose and 5.77 g⋅L^–1^ glucose) produced 1.2 mg⋅L^–1^ β-carotene (6). Because of synthesized carotenoids with different type or content, *R. glutinis* will show different color, including pink, red, or light red (7). β-Carotene, β-cryptoxanthin, torulene, and torularhodin can be produced by *R. glutinis* (8). And also, *R. glutinis* can produce some important industrial enzymes, such as phenylalanine ammonia lyase (PAL), a key enzyme in the phenylalanine pathway for the removal of ammonia from phenylalanine to produce trans-cinnamic acid (9). In addition, *R. glutinis* can achieve the purpose of improving the soil environment by adsorbing heavy metal elements (10). Even, we have screened a selenium-rich *R. glutinis* that is beneficial to the human body after converting inorganic selenate to organic selenic acid (11). Because *R. glutinis* can be cultivated from low-value raw materials and can create industrialized products through high-efficiency transformation processes, increasing researches are focusing on analyzing the reasons for its multiple applications by mining and verifying the genomic information (12, 13). The genomic data of *R. glutinis* ATCC 204091 is thought to be the first genome sequencing analysis of *R. glutinis* (14). The whole genome sequencing of *R. glutinis* WP1 was considered to be the earliest sequencing analysis to study the mechanism of mutual aid in plants by endophytic yeast (15). Through whole-genome sequencing of *R. toruloides*, lipid pathway was systematically analyzed and 150 genes that affect lipid accumulation were identified (16). Whole-genome Sequencing of *Rhodotorula* sp. CCFEE 5036 originated from Antarctica showed that a large number of genes in the metabolic pathway were positively evolved by the extreme cold environment, providing insights into the diversity of strains inhabiting polar regions (17). The omics studies of *R. mucilaginosa* revealed a positive feedback on carotenoid metabolic pathways (18). The use of genomics to investigate the function of *Rhodotorula* is a highly effective strategy.

Previously, *R. glutinis* X-20 was screened from a selenium-enriched soil on the northern slopes of the Tianshan Mountains in Xinjiang Province, China, showing a high selenium-enriched capacity with 5,349.6 μg⋅g^–1^ DWC (11). And also, it can generate large levels of β-carotene and lipids. However, the evolutionary relationship of *R. glutinis* X-20 with other *Rhodotorula* in the *Sporidiobolaceae* family is unknown. The metabolic pathways such as lipids, terpenoid biosynthesis are not well understood (19). Therefore, to reveal the evolutionary relationship of *R. glutinis* X-20 and explore its application potential in product production such as carotenoid and lipid, the whole genome was sequenced in this study. Comparative genomic analysis was then carried out to analyze the protein-coding genes of *R. glutinis* X-20. As an application case of mined functional genes from *R. glutinis* X-20, carotenoid biosynthesis pathway was heterologously expressed in *S. cerevisiae*, and carotenoid production was optimized by fermentation engineering.

## Materials and Methods

### Strain and Growth Conditions

*Rhodotorula glutinis* X-20 (CCTCC M2017225) was isolated from Shawan County (85.56E, 44.29N) on the northern slope of the Tianshan Mountains in Xinjiang, China and was preserved in the China Center for Type Culture Collection. *Rhodotorula glutinis* X-20 was incubated in conical flasks containing 100/250 mL of YPD medium (yeast extract: 10 g⋅L^–1^, peptone: 20 g⋅L^–1^, glucose: 20 g⋅L^–1^). Incubation was carried out at 30°C and 200 rpm. After 96 h cultivation, cells were centrifuged (8,000 × g, 4°C, 5 mins), frozen in liquid nitrogen for future experiments. Table 1 lists the additional strains utilized in this investigation. All strains were grown in YPD medium when necessary additional 10 mg⋅L^–1^ G418 was added. The biomass was measured using a UV spectrophotometer (UV-2600, Suzhou, Shimadzu Instruments Co., Ltd.) at 600 nm.

**TABLE 1:** Strain types.

Strain	Description	Source/References
*Saccharomyces cerevisiae INVSc1*	MATa/MAT a his341 leu2 trp1-289 ura3-52	Blazic et al. (20)
*Saccharomyces cerevisiae CEN.PK2-1C*	MATa; ura3-52; trp1-2 89; Leu2-3,112; his3 D 1; MAL2-8C; SUC2	Paramasivan et al. (21)
W8	MVA pathway enhancement	Li et al. (22)
*Sc.* CEN (B1)	*Saccharomyces cerevisiae* CEN.PK2-1C phenotype; Delta15-crtB; Delta 22-crtI	This study
*Sc.* INV (B2)	*Saccharomyces cerevisiae* INVSc1 phenotype; Delta 15-crtB; Delta 22-crtI	This study
*Sc.* CEN (B3)	*Saccharomyces cerevisiae* CEN.PK2-1C phenotype; Delta 17-crtYB; Delta 22-crtI	This study
*Sc.* INV (B4)	*Saccharomyces cerevisiae* INVSc1 phenotype; Delta 17-crtYB; Delta 22-crtI	This study
*Sc.* CEN (B5)	W8 phenotype; Delta 22-crtI	This study
*Sc.* CEN (B6)	W8 phenotype; Delta 17-crtYB	This study
*Sc.* CEN (B7)	W8 phenotype; Delta 17-crtYB; Delta 22-crtI	This study

### Genomic DNA Extraction and Sequencing

The DNA of *R. glutinis* X-20 was extracted according to the report by Lim et al. (23). DNA quality was checked by agarose gel electrophoresis and quantified using a Qubit^®^ 2.0 fluorometer (Thermo Fisher Scientific, Waltham, MA, United States). The whole genome of *R. glutinis* X-20 was sequenced at Beijing Novogene Bioinformatics Technology Co., Ltd. using the PacBio Sequel platform and the Illumina NovaSeq PE150 according to the manufacturer’s protocols.

### Genome Assembly, Prediction, and Functional Annotation

The genome was assembled using the SMRT Link v5.0.1 program. To get clean data, low quality reads (less than 500 bp) were filtered away to assure the correctness of the future analytical results. SMRT was used to fix errors, which were then combined to produce preliminary findings. The first assembly findings were compared and reviewed by comparing the reads to the assembled genome sequence in order to measure the GC content and depth of coverage of the reads in order to acquire a complete genome.

The coding genes were predicted using the program August (default parameters). RepeatMasker software (Version open-4.0.5) was used to predict repetitive elements (24). To predict gene models, RNA-seq data were aligned to the genome using the hisat2 program (25). Transcripts were reconstructed using stringtie software (version: 2.0.4), the new genes were identified in RNA-seq though not in the genome (26). The software rRNAmmer was used for rRNA prediction (27). tRNA was predicted by the Ascan-SE software (28). For sRNAs, the Rfam database was annotated first, then the final sRNA was discovered using the cmsearch program. Protein sequences of the predicted genes match from DIAMOND software (default parameters set to e-value ≤ 1e^–5^) and the highest score results (default identity ≥ 40%, coverage ≥ 40%) were selected for annotation (29). The functional annotation of predicted genes was performed using a variety of public databases, including Gene Ontology (GO), Kyoto Encyclopedia of Genes and Genomes (KEGG), Cluster of Orthologous Groups of Proteins (COG), Non-Redundant Protein Database (Nr), Transporter Classification Database (TCDB), Protein Families (Pfam), Swiss-Prot, and Carbohydrate-Active enZYmes Database (CAEZY). Secretory proteins were predicted by SignalP. Secondary metabolites were predicted using the antiSMASH program. Multiple enzymes of the cytochrome P450 family can be predicted according to the FCPD database.

### RNA Extraction and Transcriptome Sequencing

The Spin Column Fungal Total RNA Purification Kit (Sangon Biotech, Shanghai, China) was used to extract total RNA as directed by the manufacturer. RNA integrity was tested using Q-sep1 (Bioptic, New Taipei City, Taiwan China) and agarose gel electrophoresis (RNase-free). Qualified samples were subjected to DNase I digestion, followed by mRNA isolation using oligo (dT) magnetic beads, mRNA fragmentation and cDNA synthesis. “A” was added to the 3′ end of the cDNA fragment, a library adapter was attached and PCR was performed to bring up the Hybrid library. The cDNA libraries were assigned using an Illumina HiSeqTM2000 sequencer (Illumina, United States) and the raw image data files were converted to raw sequenced reads (Sequenced Reads) through Base Calling. The resulting Fastaq format files were quality controlled using the FASTQC program (version 0.11.). Clean data were obtained by cu-T adapter (version 1.11) to delete the 5′ end primer sequence and remove mismatched sequences (30). The sequence information on the comparison was obtained through the stringtie software (version: 2.0.4) to obtain the reconstructed transcript (26). To standardize the valid data, the feature Counts software (version: v1.6.0) was used to count the number of falls on the genome (31). The standardized method used in this case was FPKM (32). ANNOVAR (default parameters) software was used to annotate SNP and Indel sites. Quantitative Real-time PCR was used to assess gene expression levels. The reference gene was TUB1 and 2^–DΔ^ ^CT^ method was utilized to normalize the expression level. The One-way ANOVA method was used to do statistical analysis on all data.

### Phylogenetic Trees and the Genome Collinearity Block Analysis

The 18S rDNA sequence of *R. glutinis* X-20 was compared to other similar species in the order *Sporidiobolales* for phylogenetic analysis. The NCBI nucleotide database was used to generate all 18S rDNA sequences. MEGA 7.0 is being used to process all 18S rDNA sequences, with Clustalw (the default setting) employed for multiple sequence alignment (33). The derived glutamic acid sequencing of *R. glutinis* X-20 and closely related species were used to construct a neighbor-joining phylogenic tree. Bootstrap sampling was used with 1,000 replicates. Analysis of covariance between genomic sequences of *Rhodotorula* species was conducted out by the MCScanX (jcvi 1.1.19) program with default parameters (34).

### Comparative Genomic Analysis and Ka/Ks Calculation

Genomic data for *R. graminis* WP1, *R. taiwanensis*, and *R. toruloides* NP11 were downloaded from the NCBI database. Cluster analysis of gene families was carried out using OrthoFinder 2.5.4. AgBase-GOanna database was used for GO annotation with default parameters. Statistical annotation results were then plotted using the R language ggolot2 package. As the NG model, the Ka/Ks Calculator 2.0 model was used to estimate Ka/Ks values for each single copy gene trio.

### Cloning and Functional Analysis of Target Genes

The Primer 5 software was used to design primers for the crtI (GenBank Accession: OL518983) and crtYB (GenBank Accession: OL518982) genes derived from *R. glutinis* X-20. Conserved domain analysis was performed by CD-search^1^ on NCBI website. Secondary structure analysis was performed using Secondary Structure Prediction Server, while molecular masses and theoretical isoelectric points were also performed using Expasy for preliminary prediction of function. The gene expression cassettes crtI and crtYB were constructed to further validate their expression in *S. cerevisiae*.

### Production of Carotenoids in *Saccharomyces cerevisiae* by Expressing crtI and crtYB

The genes of crtI and crtYB were amplified from the cDNA of *R. glutinis* X-20. The crtB (GenBank Accession: KC954270.1) originated from *Erwinia* was chemically synthesized. The promoters (FBA1p, ALA1p, and TEF1p) and terminators (ADH1t, HOG1t, and CYC1t) were amplified from *S. cerevisiae* genome. The gene expression cassettes TEF1p-crtB-CYC1t, ALA1p-crtI-HOG1t, and FBA1p-crtYB-ADH1t were constructed by OE-PCR. The crtB, crtI, and crtI gene expression cassettes were co-transferred into *S. cerevisiae* by the lithium acetate method (35). Positive strains were obtained by colony PCR and sequencing. The engineered strain was fermented in culture medium at 200 rpm and 25°C to produce carotenoids.

### Extraction and Identification of Metabolites

For the analysis of metabolites, approximately 60 mg of cells were obtained by centrifuging (8,000 × g, 4°C, 10 mins). Then, 500 μL of methanol (−20°C) and 500 μL of H_2_O (4°C) were added into a 2 mL tube containing cells. After vortexing for 30 s, 100 mg of glass beads were added, and immersed in liquid nitrogen for 5 mins. Samples were agitated in a grinder at 55 Hz for 2 mins and centrifuged (8,000 × g, 4°C, 10 mins). The supernatant was extracted, concentrated, and dried. To acquire prepared samples for LC-MS, the sample was dissolved by 300 μL of 2-chlorophenylalanine (4 ppm) methanol aqueous solution (1:1, 4°C) and the supernatant was aspirated and filtered through 0.22 μm filter membrane. For HPLC-MS analysis, an ACQUITY UPLC^®^ HSS T3 (150 × 2.1 mm, 1.8 μm, Waters) column with an electrospray ionization source (ESI-MSn) was used to separate and identify the metabolites of extracts, as determined by Monnerat et al. (36). Lycopene and β-carotene were determined according to the report by Li et al. (37).

### Statistics and Analysis

The experimental data were replicated three times and the data are presented as the average of three independent experimental samples with mean ± SD (*n* = 3). To evaluate if there was a statistically significant difference between the group data, the One-way ANOVA method was utilized.

## Results

### Assembly Features of *Rhodotorula glutinis* X-20

**FIGURE 1:**
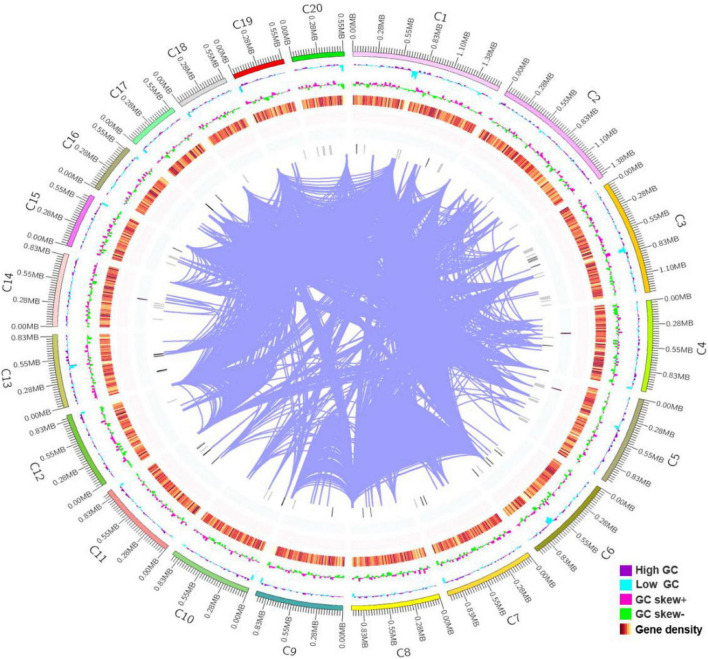
Genomic features of *Rhodotorula glutinis* X-20. The 21.6 Mb genome of *R. glutinis* X-20 contains 20 scaffolds (the longest length scaffold of 1,634,659 bp, a N50 length of 913,949 bp, sum of Contig Lengths of 21,850,050 bp, GC content of 68.21%). The *R. glutinis* X-20 genome encodes 6,892 predicted proteins and 157 transfer RNAs (tRNAs), which are validated by RNA-seq. From the outer circle to the inner, it represents the length of scaffolds, GC content is higher than the average (purple), GC content is lower than the average (blue), GC skew curve (Pink: positive GC skew; Green: negative GC skew) and the gene density of coding genes, rRNA, snRNA, tRNA (gradient color), the darker the color, the greater the gene density in the window and chromosome duplication, respectively.

The whole genome shotgun strategy of *R. glutinis* X-20 was carried out utilizing a PacBio Sequel system with a single molecule real time (SMRT) sequencer and an Illumina NovaSeq PE150 system. In total, 2.95 Gb of polymerase reads were generated. To calculate the genome size of *R. glutinis* X-20, we computed the overall 15-mer number to be 650,302,860 bp and the k-mer depth to be 28.27 Mb. The estimated genome size of *R. glutinis* X-20 was calculated to be 23 Mb using formula for 15-mer depth frequency distribution. Based on the genome information of proximate yeast, according to the genome size of about 20 M, the depth of this sequencing was probably more than 100× (38). The assembled genomic sequences were merged with the expected coding gene findings, and the sample genomes were displayed using Circos software (39). As shown in Figure 1, the genome of *R. glutinis* X-20 was finally assembled by organizing the contigs larger than 500 kb from longest to smallest, corresponding to 20 contigs in order, the longest scaffold is 1,634,659 bp, N50 length is 913,949 bp, GC content is 68.21%, and the sum of Contig Lengths is 21,850,050 bp, with a size of 21.6 Mb. A total of 6892 genes were projected from scratch by Augustus software, with an average length of 1,813 bp and an average GC content of 69.59%, representing 55.0% of the whole genome sequence. Tandem repeat sequences can be obtained by genome sequencing as an indicator of species genetic characteristics and evolutionary relationships. TRF (Tandem Repeats Finder, Version 4.07b) (40) detected 6,617 tandem repeats (TR), including 4,555 minisatellite DNA and 1,255 microsatellite DNA, accounting for 4.56% of the genomic sequence. The integrity of gene assembly was assessed by BUSCOs (41). The integrity assessment of BUSCOs discovered that the assembled genome comprised 735 intact BUSCOs (97.0%), 733 in single-copy, and 2 in duplicated form. The RNA-seq results revealed that 6,822 (98.98%) of the genes predicted for the *R. glutinis* X-20 genome regions were expressed, and 441 new genes were identified. These findings revealed that the assembled genome included a sizable proportion of the entire number of genes. The BUSCOs calibrations and transcript sequencing both demonstrated that our present genomic assemble was of good quality.

### Functional Annotation

The predicted gene protein sequences were compared to each functional database using DIAMOND (E-value < 1e^–5^). First, annotation using Nr database indicated that 6,547 (94.99%) of the 6,892 predicted genes have been annotated on 29 different species. Based on the distribution of the number of annotations, the top three strains were *Rhodotorula graminis* (5,379, 82%), *Rhodotorula toruloides* (899, 13.7%), and *Rhodotorula taiwanensis* (104, 1.58%). According to the KEGG, GO, TCDB, Swissport, and Pfam databases, 3,584 (52.00%), 4,558 (66.13%), 248 (3.6%), 1,917 (29.28%), and 4,558 (69.62%) genes could be annotated. Furthermore, the antiSMASH software, which analyzes secondary metabolites, identified 70 genes that express six clusters (three terpene, one NRPS, and two others). To study the metabolic pathways of gene products and compounds, a total of 3,584 genes were effectively mapped to 354 KEGG pathways (Supplementary Figure 1). Furthermore, 4,558 genes were predicted to classify into three Gene Ontology (GO) categories (Supplementary Figure 2). Cellular processes and metabolic processes accounted for 28.25 and 26.13%, respectively. Porters (uniporters symporters antiporters) (92, 37.09%), P–P-bond-hydrolysis-driven transporters (65, 26.21%), and Oxidoreduction-driven transporters (29, 11.69%) were the top three membrane transport proteins studied from the 248 genes identified in the TCDB database. These indicate that its transport proteins pathway has been strengthened.

### Analysis of Phylogenetic and Syntenic Relationships

**FIGURE 2:**
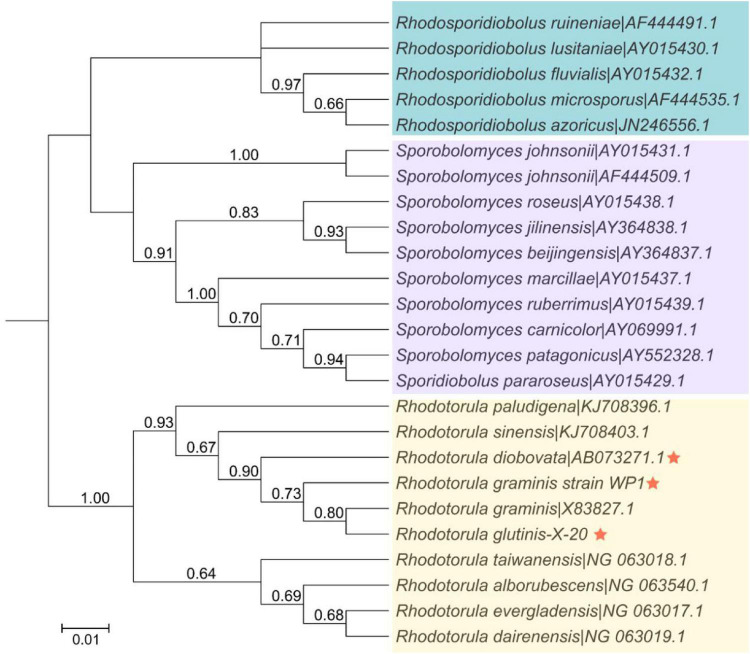
Phylogenetic and evolutionary analysis between 25 strains of red yeasts of the order *Sporidiobolales*. The phylogenetic tree was built in MEGA 7.0 using an alignment of the 18S rDNA sequences, the Neighbor-joining technique, and a Bootstrap analysis of 1,000 iterations. An asterisk denotes the strain *R. glutinis* X-20 and its closely related species. The bootstrap value is shown by the number at each branch of the phylogenetic tree (1,000 replicates). Shades of blue, purple, and yellow represent the various clusters of *Rhodosporidiobolus*, *Sporobolomyces*, and *Rhodotorula* isolates, respectively.

**FIGURE 3:**
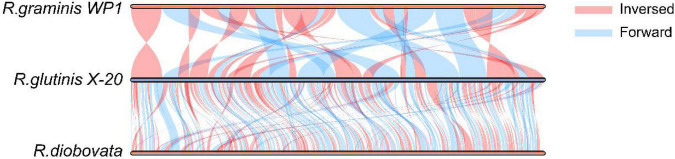
Analysis of the genome collinearity blocks between *R. glutinis* X-20 genes with its two close related species. MCScanX was used to conduct pair-wise alignments of *R. glutinis* X-20, *R. graminis* WP1, and *R. diobovata* complete genome sequences. In three genomes, blue lines represent Forward collinear blocks and pink bars represent Inversed collinear blocks.

The relationship of *R. glutinis* X-20 to other red yeast species was investigated by constructing phylogenetic trees from three directly homologous *Sporobolomyces* families (*Rhodosporidiobolus*, *Rhodotorula*, and *Sporobolomyces*). The 18sDNA sequence of *R. glutinis* X-20 was compared with the 18s rDNA sequences of other strains (data from the NCBI nucleotide database). As shown in Figure 2, the evolutionary tree demonstrated that *R. glutinis* X-20 is more closely related to *R. graminis*, *R. graminis* WP1, and *R. diobovata* than other species, supporting the annotated results in the Nr database. The genome collinearity blocks of *R. glutinis* X-20 and its two accessible *R. graminis* WP1 and *R. diobovata* genomes were examined using phylogenetic tree results and genome sequences from the NCBI website. As shown in Figure 3, there are 38 blocks (6,343 synthetic gene pairs) between the *R. glutinis* X-20 genome and the *R. graminis* WP1 genome sequence. The number of genes was 92.03% for *R. glutinis* X-20 and 89.79% for *R. graminis* WP1. Between the *R. glutinis* X-20 genome and the *R. diobovata* genome sequence, there are 350 blocks (6,086 synthetic gene pairs). The number of genes in *R. glutinis* X-20 and *R. diobovata* was 88.31 and 79.36%. The complete length of collinearity blocks accounted for 20.26% of the genome length of *R. glutinis* WP1 and 63.70% of the genome length of *R. diobovata*, in both. These findings imply that the *R. glutinis* X-20 genome is more closely in linked to the *R. graminis* WP1 genome than the *R. diobovata* genome, and that the *R. glutinis* X-20 genome is better annotated.

### Protein Families and Comparative Genomes

**FIGURE 4:**
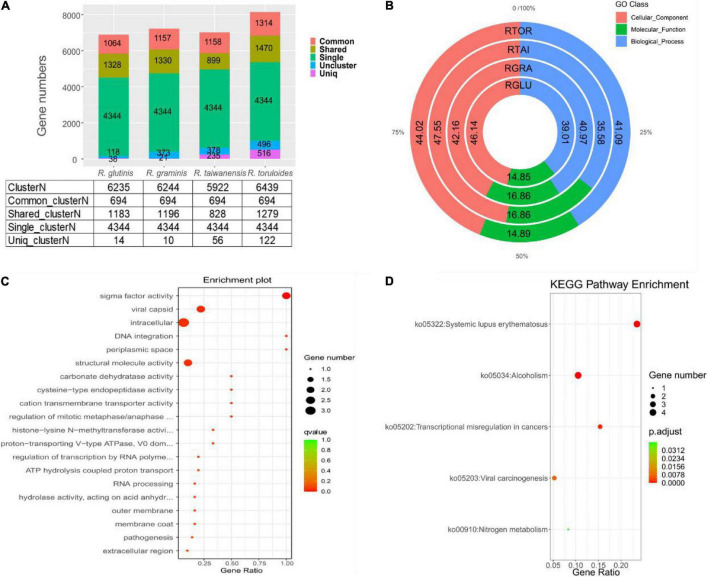
*Rhodotorula glutinis* X-20 and closely related *Rhodotorula* strains such as *R. graminis* WP1, *R. taiwanensis*, and *R. toruloides* NP11 were used for comparative genomic analysis. **(A)** Gene family analysis on four red yeast strains. **(B)** The relative proportions (%) of Uniq geneN enrichment in four red yeast species in GO categories. **(C)** Top 20 enriched GO annotations of these species-specific genes in *R. glutinis* X-20 whole genome. **(D)** Enrichment of the Uniq gene N in *R. glutinis* X-20 by the KEGG pathway. The rich factor is the ratio of the number of enriched genes to the total number of genes in a given pathway. The Q-value is derived from the p-value after multi-test correction. Q-values vary from 0 to 1, with the closer to 0 indicating greater enrichment.

For the 6892 protein-coding genes predicted in *R. glutinis* X-20, the top three predicted *Rhodotorula* strains in the NR database, *R. graminis* WP1, *R. taiwanensis*, and *R. toruloides* NP11, were selected for functional gene analysis of homologous strains. The protein sequences of four *Rhodotorula* were analyzed by cluster the data of gene families separately using OrthoFinder 2.5.4 (default parameters). By cluster analysis, 6951 protein cluster families (6235 cluster of *R. glutinis* X-20, 6244 cluster of *R. graminis* WP1, 5922 cluster of *R. taiwanensis*, and 6439 cluster of *R. toruloides* NP11) and 4486 shared_cluster (1183 shared_cluster of *R. glutinis* X-20, 1196 shared_cluster of *R. graminis* WP1, 828 shared_cluster of *R. taiwanensis*, and 1279 shared_cluster of *R. toruloides* NP11) were identified, while 694 common_cluster families and 4344 single_cluster gene families were identified (Figure 4A). 14 Uniq clusterN (38 genes), 10 Uniq clusterN (21 genes), 56 Uniq clusterN (235 genes), and 122 Uniq clusterN (516 genes) protein families are Uniq clusterN (Uniq geneN) in *R. glutinis* X-20, *R. graminis* WP1, *R. taiwanensis*, and *R. toruloides* NP11, respectively. In addition, in order to understand the functional categorization of Uniq geneN in *R. glutinis* X-20, specific genes in each of the four red yeast species were subjected to GO analysis. As shown in Figure 4B, 46.14% of the cellular component, 39.01% of the biological process and 14.85% of the molecular function were identified in *R. glutinis* X-20. *R. glutinis* X-20 functional distribution is more oriented toward cellular component. 10, 18, and 7 GO terms in Uniq_geneN of *R. glutinis* X-20 were significantly enriched in three categories of biological processes (BP), molecular functions (MF), and cellular components (CC), respectively (Figure 4C). According the results, Uniq geneN and transmembrane function were related, including MF: sigma factor activity (GO:0016987), structural molecule activity (GO: 0005198), carbonate dehydratase activity (GO:0004089), cysteine-type endopeptidase activity (GO:0004197), cation transmembrane transporter activity (GO:0008324); CC: viral capsid (GO:0019028), intracellular (GO:0005622), periplasmic space (GO:0042597); BP: DNA integration (GO:0015074), regulation of mitotic metaphase/anaphase transition (GO:0030071). Finally, Uniq geneN was counted through the KEGG database. The KEGG pathway that shows to be enriched in *R. glutinis* X-20 Uniq geneN comprises primarily nitrogen metabolism (ko00910), viral carcinogenesis (ko05203), transcriptional misregulation in cancers (ko05202), alcoholism (ko05034), systemic lupus erythematosus (ko05322) (Figure 4D). This shows that *R. glutinis* X-20 may be pathogenic as well. The assessment of Ka/Ks for single-copy orthologous genes is generally accepted as an indicator of selection pressure during biological evolution. The 4344 single-copy cluster family between *R. glutinis* X-20 and three of his related strains was used as a proxy to calculate the Ka/Ks ratio in order to investigate the evolution of the target strains. The substitution rate (Ka/Ks) for each orthologous gene was calculated using the NG model. There were three pairs with Ka/Ks values greater than 1.0 (strong positive selection), 114 pairs with Ka/Ks values between 0.5 and 1.0 (positive selection), 3,204 pairs with Ka/Ks values between 0.1 and 0.5 (weak positive selection), and 1,015 pairs with Ka/Ks values less than 0.1 (purifying selection). Only three gene pairs with Ka/Ks > 1 in *R. glutinis* X-20 were significant, suggesting that the most of genes were preserved by a sequence of purifying natural selections. By KEGG and GO annotation of purifying selected genes (Ka/Ks > 0.1), *R. glutinis* X-20 is believed to have a strong evolutionary tendency in metabolite biosynthesis as well as cell division function (Supplementary Figures 3, 4).

### Metabolites Identification in *Rhodotorula glutinis* X-20

To further analyze the function of the *R. glutinis* X-20 strain from a metabolite point of view, metabolites in samples obtained after 120 h of growth were analyzed using HPLC-MS for qualitative and quantitative characterization (42). In total, 654 species were detected by scanning in data-dependent MS/MS mode, mainly including 171 amino acid, 65 carbohydrate, 32 cofactors and vitamins, 124 lipid, 44 nucleotide, 4 peptide, and 214 others (Table 2). The important lipids were identified, including 40.14% fatty acyl, and 8.45% prenol lipids. This suggests that *R. glutinis* X-20 is a typical lipid-producing yeast strain, producing mainly fatty acyl and prenol lipids (Table 3). Further analysis of lipids found several more important compounds such as linolenate, methyl palmitate, β-carotene, and L-phenylalanine (Supplementary Table 4). Linoleic and methyl palmitate, the main lipid compounds produced by *R. glutinis* have a positive impact on softening blood vessels and lowering blood pressure in humans. β-carotene and L-phenylalanine are two of the most widely studied substances in *R. glutinis*, with considerable industrial applications.

**TABLE 2:** Identified metabolites using HPLC-MS profiling in *Rhodotorula glutinis* X-20.

Metabolites	Positive	Negative	Percentage (%)
Amino acid	113	58	26.14
Carbohydrate	29	36	9.94
Cofactors and Vitamins	21	11	4.89
Lipid	83	41	18.96
Nucleotide	30	14	6.73
Peptide	2	2	0.61
Others	170	44	32.72
Total	448	206	100

**TABLE 3:** Identified lipids using HPLC-MS profiling in *Rhodotorula glutinis* X-20.

Lipids	Positive	Negative	Percentage (%)
Azacyclic compounds	1	1	1.40
Benzene and substituted derivatives	1	–	0.70
Carboxylic acids and derivatives	5	1	4.22
Fatty Acyls	37	20	40.14
Flavonoids	4	1	3.52
Glycerophospholipids	2	1	2.11
Hydroxy acids and derivatives	1	1	1.40
Isoflavonoids	–	1	0.70
Keto acids and derivatives	2	–	1.40
Organic oxides	–	1	0.70
Organic phosphoric acids and derivatives	–	1	0.70
Organonitrogen compounds	–	3	2.11
Organooxygen compounds	1	1	1.40
Oxanes	1	–	0.70
Polycyclic hydrocarbons	3	–	2.11
Prenol lipids	11	1	8.45
Pyrans	1	–	0.70
Pyrimidine nucleotides	1	–	0.70
Steroids and steroid derivatives	6	5	7.74
Stilbenes	1	–	0.70
Unsaturated hydrocarbons	1	–	0.70
Others	22	3	17.60
Total	101	41	100

### Validation of Putative Phytoene Desaturase and Putative 15-Cis-Phytoene Synthase/Lycopene Beta-Cyclase

On the basis of whole genome sequencing, nucleotide primers crtI-F and crtI-R were constructed, as well as crtYB-F and crtYB-R, to clone the putative phytoene desaturase gene (crtI) and the putative 15-cis-phytoene synthase/lycopene beta-cyclase gene (crtYB) from *R. glutinis* X-20 (Supplementary Table 1). The CDS region encoding the crtI sequence is composed of a full-length ORF region and six shorter ORF sections, 1,644 bp long (containing 547 amino acids) (Supplementary Table 2). Molecular weight, Isoelectric point, Domain, Protein Family, Secondary structure of crtI and crtYB genes were statistics (Table 4).

**TABLE 4:** Functional analysis of crtI and crtYB.

Type	crtI	crtYB
Sequence length	1,644 bp (547 amino acid)	1,776 bp (591 amino acid)
Molecular weight	60.84 kDa	65.39 kDa
Isoelectric point	7.35	6.3
Domain	crtI	CarR; Isoprenoid biosynthesis enzyme
Protein Family	crtI subfamily member	cl11889 super family; Isoprenoid BiosynC1 super family
Secondary structure	(H): 75.5%, (E): 64.6%, (T): 11.1%	(H): 76.6%, (E): 45.7%, (T): 10.5%

### Expression of crtI and crtYB in *Saccharomyces cerevisiae*

**FIGURE 5:**
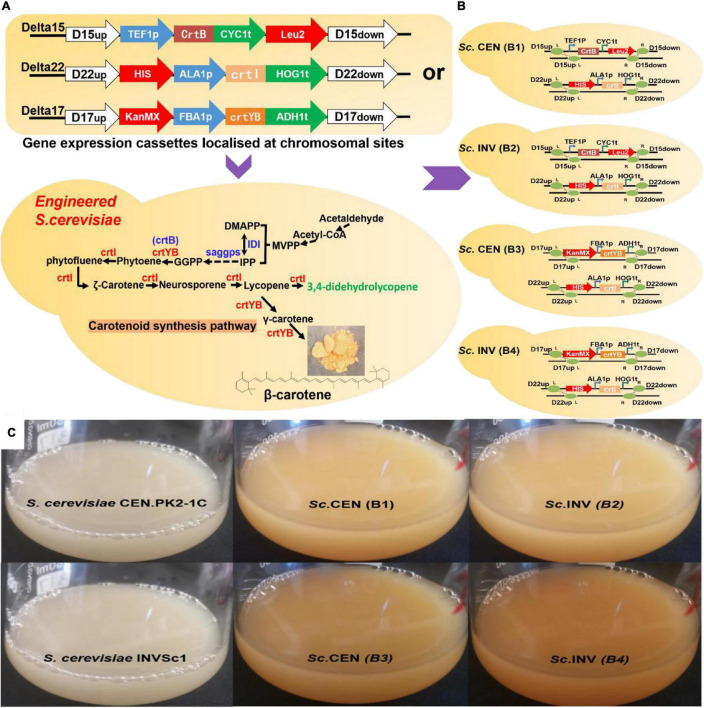
Validation of gene function in *Saccharomyces cerevisiae*. **(A)** Gene expression cassettes are constructed and transferred to chromosomal locations in *S. cerevisiae* by homologous recombination methods. **(B)** Specific genotype of engineered strains. *Sc*. CEN (B1), *Sc*. INV (B2), *Sc*. CEN (B3), and *Sc*. INV (B4). **(C)** Picture after 118 h of shake-flask fermentation: *S. cerevisiae* CEN.PK2-1C, *S. cerevisiae* INVSc1, *Sc*. CEN (B1), *Sc*. INV (B2), *Sc*. CEN (B3), and *Sc*. INV (B4).

To detect the putative phytoene desaturase (crtI) in *R. glutinis* X-20, the gene of phytoene synthase (crtB) from *Erwinia* (GenBank Accession: KC954270.1) was cloned to provide the necessary supply of precursors. The gene expression cassettes TEF1p-crtB-CYC1t and ALA1p-crtI-HOG1t were first homologously transferred to wild *S. cerevisiae* chromosomes Delta 15 and Delta 22, respectively (Figure 5A). The shake flask fermentation revealed that the strains with the properly modified gene expression cassette changed color from white to pink, whereas the control showed no color change (Figure 5C). HPLC analysis showed that the accumulated carotenoids in the hybrid strains containing TEF1p-crtB-CYC1t and ALA1p-crtI-HOG1t were identical with the standard lycopene, showing that the crtI gene functions as a multi-step phytoene desaturase (Supplementary Figure 7). In addition, to test the effect of different chassis hosts on the products, crtI and crtB were inserted into *S. cerevisiae* CEN.PK2-1C (*Sc*. CEN) and *S. cerevisiae* INVSc1 (*Sc*. INV) to construct strains *Sc*. CEN (B1) and *Sc*. INV (B2), respectively (Figure 5B).

To assay the activity of the putative 15-cis-phytoene synthase/lycopene beta-cyclase (crtYB) from *R. glutinis* X-20, the gene expression cassettes FBA1p-crtYB-ADH1t and ALA1p-crtI-HOG1t were similarly constructed into Delta 17 and Delta 22, respectively, on the chromosome of wild *S. cerevisiae* (Figure 5A). The fermented strains show a change from white to yellow in the transformed strains and no change in the control (Figure 5C). HPLC analysis showed that the accumulated carotenoids in the hybrid strains containing gene expression cassettes ALA1p-crtI-HOG1t and FBA1p-crtYB-ADH1t were identical with the standard β-carotene, showing that the crtYB gene functions as 15-cis-phytoene synthase/lycopene beta-cyclase (Supplementary Figure 7). Meanwhile, crtYB and crtI were co-introduced into the different chassis hosts *S. cerevisiae* CEN.PK2-1C and *S. cerevisiae* INVSc1 to construct strains *Sc*. CEN (B3) and *Sc*. INV (B4) (Figure 5B).

**FIGURE 6:**
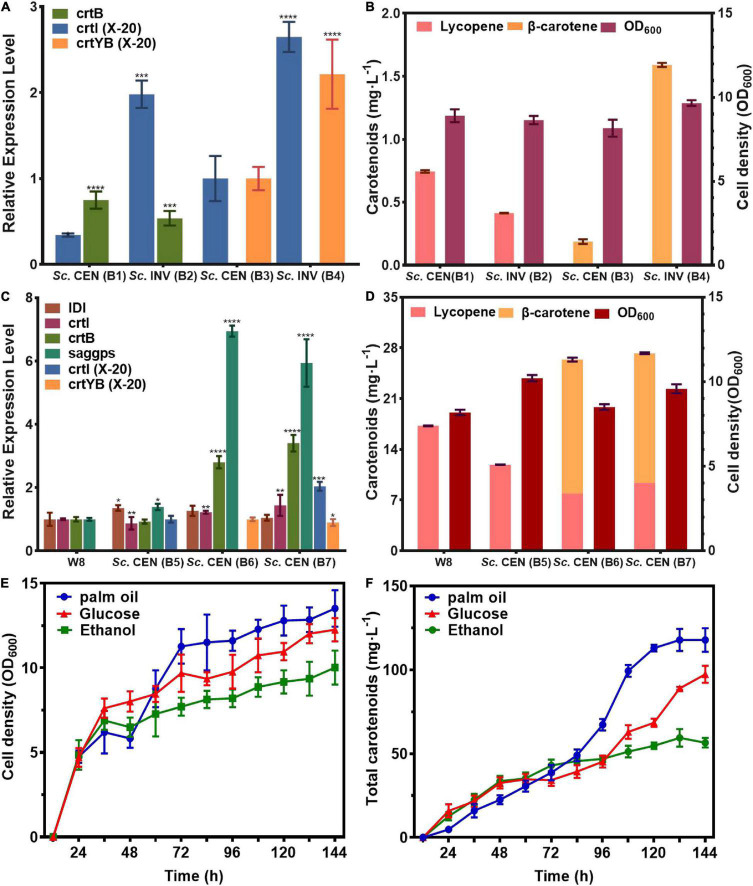
Efficient expression and fermentation optimization of engineered strains. Validation of genetically functional engineered strains for **(A)** mRNA expression levels, **(B)** metabolite production. Optimization of **(C)** mRNA expression levels of genes of interest in the upstream pathway for engineered strains with **(D)** metabolite production. **(E)** Cell growth density of *Sc*. CEN (B7) strain, **(F)** total carotenoids production of *Sc*. CEN (B7) strain (relative mRNA levels were calculated from the mRNA of the reference gene TUB1, data shown as the mean of three independent experiments, error expressed as ±SD). Significant differences between groups were calculated by one-way ANOVA (****p < 0.001, ***p < 0.01).

The qPCR showed the effective transcription of crtI, crtB genes in *Sc*. CEN(B1), *Sc*. INV (B2), and crtI, crtYB genes in *Sc*. CEN (B3), *Sc*. INV (B4). Besides, compared to *Sc*. CEN(B3), mRNA levels of both crtI and crtYB genes were boosted in strain *Sc*. INV(B4), and compared to mRNA levels of crtB in *Sc*. CEN(B1) and *Sc*. INV(B2), mRNA levels of crtYB genes in *Sc*. CEN(B3) and *Sc*. INV(B4) were increased 1.87- and 4.12-fold (Figure 6A).

This effect was more pronounced when crtI and crtYB were co-expressed. These results indicated that the crtI, crtYB genes were fully functional in *S. cerevisiae*, and the mRNA level of the crtYB gene in *S. cerevisiae* was increased compared to the mRNA level of crtB in *S. cerevisiae*. After 118 h of shake-flask fermentation, HPLC analysis were used to determine the products (Figure 6B). HPLC analysis showed that the engineered strain *Sc*. CEN (B2), constructed with *S. cerevisiae* CEN.PK2-1C as the chassis host, produced lycopene at a titer of 0.74 mg⋅L^–1^ (0.36 mg⋅g^–1^ DCW) and the engineered strain *Sc*. INV (B1), constructed with *S. cerevisiae* INVSc1 as the chassis host, produced lycopene at a titer of 0.41 mg⋅L^–1^ (0.17 mg⋅g^–1^ DCW). In comparison, *Sc*. CEN (B2) was 1.8 folds higher in titer than *Sc*. INV (B1). Similarly, when the engineered strain *Sc*. CEN (B3), constructed with *S. cerevisiae* CEN.PK2-1C as the chassis host, produced β-carotene at a titer of 0.18 mg⋅L^–1^ (0.18 mg⋅g^–1^ DCW), the engineered strain *Sc*. INV (B4), constructed with *S. cerevisiae* INVSc1 as the chassis host, produced β-carotene at a titer of 1.59 mg⋅L^–1^ (0.63 mg⋅g^–1^ DCW). The 8.8-fold increase in the titer of *Sc.* INV (B4) suggests that *S. cerevisiae* INVSc1 is better suited as a chassis host for crtI and crtYB gene expression. This finding is consistent with prior findings by Westfall et al. such as the production of artemisinin by *S. cerevisiae* (43).

### Carotenoids Production in *Saccharomyces cerevisia*e by Expressing crtI and crtYB

The rate-limiting step in carotenoid biosynthesis is the mevalonate pathway, and GGPP is the key rate-limiting enzyme (44). To further clarify the expression effect of genes from *R. glutinis* X-20, the mevalonate pathway in *S. cerevisiae* was fortified for further functional analysis of crtI and crtYB. crtI, crtYB or crtI-crtYB were transferred to previously constructed mevalonate pathway optimized the engineered strain W8 (22), produced *Sc*. CEN (B5), *Sc*. CEN (B6), and *Sc*. CEN (B7). The results of qPCR showed the effective transcription of the crtI and crtYB in engineered yeasts (Figure 6C). Significant transcription of the crtI gene in *Sc.* CEN (B5), the rate-limiting enzyme IDI of the MVA pathway, and saggps of the carotenoid synthesis pathway both were up-regulated in *Sc*. CEN (B5) compared to the control engineered strain W8 (Figure 6C). The control strain W8 produced 17.25 mg⋅L^–1^ (2.78 mg⋅g^–1^ DCW), but the lycopene production of strain *Sc*. CEN (B5) was reduced to 11.89 mg⋅L^–1^ (1.99 mg⋅g^–1^ DCW) (Figure 6D). This may be due to further conversion of the crtI gene to 3,4-didehydrolycopene, causing further expression of the upstream rate-limiting enzyme and at the same time consumption of lycopene (45). In the *Sc*. CEN (B6) strain, IDI, saggps, crtB and crtI were also found up-regulated with 1. 26-, 6. 94-, 2. 8-, and 1.22-fold, respectively, probably caused by dragging *via* overexpression of the crtYB gene (Figure 6C). *Sc*. CEN (B6) produced 7.91 mg⋅L^–1^ (1.25 mg⋅g^–1^ DCW) lycopene and 18.48 mg⋅L^–1^ (2.92 mg⋅L^–1^ DCW) β-carotene, a 0.66-fold decrease in lycopene production compared to *Sc*. CEN (B5), which newly accumulated high concentrations of β-carotene (Figure 6D). Furthermore, strain *Sc*. CEN (B7) including crtI and crtYB showed a 5.93-fold upregulation of saggps, a 1.44-fold upregulation of crtI and a 3.4-fold upregulation of crtB compared to the control engineered W8 strain (Figure 6C). Expression of crtI and crtYB resulted in a 0.78-fold decrease in lycopene 9.37 mg⋅L^–1^ (1.47 mg⋅L^–1^ DCW) compared to *Sc.* CEN (B5), but production 17.89 mg⋅L^–1^ (2.80 mg⋅L^–1^ DCW) of β-carotene (Figure 6D). These results indicated that crtI and crtYB can be efficiently expressed in *S. cerevisiae*.

In order to obtain high levels of carotenoid production, the *Sc*. CEN (B7) strain was used in 1L bioreactors (TJ-Mini Box, Parallel-Bioreactor, Shanghai) for carotenoid fermentation. The initial glucose was 20 and 2 g⋅L^–1^ palmitic acid, 2 g⋅L^–1^ glucose, and 2 g⋅L^–1^ ethanol was added after 24 h of fermentation. Sodium hydroxide/sulfuric acid was used to maintain pH at 6.0 during fermentation. The addition of palmitic acid well increased the cell growth (46). Supplementing palmitic acid resulted in a faster cell growth 48–72 h compared to the addition of glucose or ethanol (Figure 6E). Total carotenoids (β-carotene, lycopene) production gradually increased throughout the fermentation, with glucose and ethanol titers of 97.29 and 64.97 mg⋅L^–1^, respectively, and 117.78 mg⋅L^–1^ under palmitic acid conditions (Figure 6F). With the addition of palmitic acid, the titers of lycopene and β-carotene were 53.55 and 64.23 mg⋅L^–1^, respectively, with a 5.75- and 3.59-fold rise in supplementary fermentation, respectively.

## Discussion

The functional genes of *R. glutinis*, an oil-producing yeast, are used to produce a range of metabolites. Researchers are increasingly considering *R. glutinis* as a potential strain for industrial applications (47). Unfortunately, several *R. glutinis* have been described in the literature for the commercial production of lipids and carotenoids, but there are few validation studies on their molecular biology and functional genes. As a result, genomic analysis enables more in-depth research of its function. Furthermore, it is valuable that the engineered strains are used and perfectly expressed by key functional genes.

The functional resolution of the *R. glutinis* X-20 genome and metabolite assay showed a substantial number of lipid metabolism genes, as well as a number of other relevant genes. The results of the whole genome assembly were classified according to their valuable functional genes and a phylogenetic tree was performed to analyse their evolutionary position in the family. *R. graminis* WP1 and *R. diobovata* were identified as strains with the highest affinity, and analysis of genome collinearity blocks revealed that *R. glutinis* X-20 was better assembled. Three direct homologous *Rhodotorula* families were selected for comparative genomic analysis to better investigate its gene function. The gene function of *R. glutinis* X-20 was shown to be prominently distributed in functions related to transmembrane transport. According to the KEGG annotation, *R. glutinis* X-20 has a number of disease-causing genes that are consistent with those revealed for Wirth et al. (2). Though Ka/Ks analysis, *R. glutinis* X-20 has a strong evolutionary tendency in metabolite biosynthesis as well as in cell division functions.

*Rhodotorula glutinis* is a lipid-producing strain which can synthesize a wide range of lipids in the body (48). *Rhodotorula glutinis* synthesizes acylglycerols, fatty acids, phospholipids and sterols in general, which are essential for cellular life (49). The typical method involves overexpression of important enzymes, suppression of competing pathways, and strain adjustment of the transcription pathway of the relevant genes (48). Comparison with the gene annotation results identified a number of functionally significant genes. Such as GPD1, FAS1, ACACa, ACOX3, TGL2, MGLL, and PDAT. As a case, a valuable phenylalanine ammonia lyase (PAL) was also identified, which has been used as an enzyme-based biopharmaceutical therapy for the treatment of phenylketonuria (PKU) (50). A number of genes associated with fatty acid synthesis were identified, as these lipases were closely related to lipid metabolism (51). With the further exacerbation of energy crisis in the face of both global epidemics and economic pressures, biofuels production by *R. glutinis* maybe more advantageous as the third generation than first and second generation biofuels (52). Therefore, it is of great significance to study the lipid metabolism of *R. glutinis* X-20.

When metabolites were analyzed by HPLC-MS, a variety of carotenoid species, including canthaxanthin, astaxanthin, and β-carotene were identified. The crtI and crtYB genes are associated with the formation of β-carotene. This was consistent with the production of β-carotene by *R. glutinis* ATCC 15125 reported by Braunwald et al. (53). As the most important carotenoid in *R. glutinis*, β-carotene is unique in that it acts as a precursor to vitamin A, which is converted to vitamin A in the body after consumption and is used in the treatment of night blindness, skin diseases, bronchitis, and chronic pharyngitis (54, 55). However, the construction of engineered strains for efficient carotenoid synthesis is still lacking, especially with *R. glutinis* as the original host. In order to achieve commercial application of β-carotene, it is essential to obtain efficient *R. glutinis* strains, but there are not strains of *R. glutinis* for industrial application so far. The main reasons for this are the high cost of the culture medium, the complexity of the operation and the low yield per unit volume of the culture vessel, among other disadvantages that make it unsuitable for industrial production (56). Furthermore, *Rhodotorula* has previously been reported to cause fungemia associated with central venous catheter (CVC) use, which may be the reason why this strain is not suitable for direct use as an industrial strain. The use of synthetic biology approaches could therefore enable us to establish a strain with high β-carotene production by heterologously expressing the relevant genes in a safe and healthy strain. Due to its depth of research and safety, *S. cerevisiae* is widely used as a gene expression host (57). Thus, the crtI and crtYB key genes in *R. glutinis* X-20 were cloned and constructed onto chromosomes in *S. cerevisiae*. As shown in Figure 6, crtI can synthesize lycopene, and the crtYB is bifunctional which can synthesizes phytoene and β-carotene. This was consistent with what was previously reported by Li et al. (58). In addition, in comparison to crtB, crtI, and crtYB were more highly expressed in *S. cerevisiae* INVSc1. This indicated that the genetic background has an influence on the expression of the target enzymes (59). Co-expression of crtI and crtYB in *S. cerevisiae* INVSc1 produced 1.59 mg⋅L^–1^ (0.63 mg⋅L^–1^ DCW) of β-carotene. Compared with the previous results that transferred crtE, crtYB, and crtI from *Phaffia rhodozyma* into *S. cerevisiae* to produce 258.8 μg⋅g^–1^ DWC (60), the yield of co-expressed crtI and crtYB was increased by 2.45-fold.

To explore the application potential of crtI and crtYB, they were transferred into strain W8, in which the mevalonate pathway had been strengthened (22, 44). When the crtI gene was introduced alone, the rate-limiting enzyme of the upstream MVA pathway was up-regulated 1.39-fold, but the *Sc.* CEN (B5) strain produced 0.31-fold less lycopene. This suggests that crtI possesses a further conversion to 3,4-didehydrolycopene, leading to further expression of the upstream rate-limiting enzyme and concomitant depletion of lycopene. When the crtYB gene was introduced alone, HPLC showed that *Sc*. CEN (B6) produced 7.91 mg⋅L^–1^ (1.24 mg⋅g^–1^ DCW) of lycopene and 18.48 mg⋅L^–1^ (2.92 mg⋅g^–1^ DCW) of β-carotene compared to W8, when the transcript levels of the key upstream rate-limiting enzyme saggps were up-regulated by 6.94-fold. This was consistent with Rathod et al. who showed that the introduction of crtYB (phytone-β-carotene) from the red yeast *Xanthophyllomyces dendrorhous* into *Chlamydomonas reinhardtii* to overexpress lycopene synthase, this was consistent with the production of β-carotene (22.8 mg⋅L^–1^) and lutein (8.9 mg⋅L^–1^), suggesting that Sc. CEN(B6) itself can consume some precursors to produce β-carotene (61). When crtI and crtYB were co-expressed, 9.37 mg⋅L^–1^ (1.47 mg⋅L^–1^ DCW) lycopene and 17.89 β-carotene mg⋅L^–1^ (2.80 mg⋅L^–1^ DCW) were produced in *Sc*. CEN (B6). Lycopene rose 0.18-fold and β-carotene remained essentially unchanged, indicating that the crtYB gene had reached its highest level of expression. This further validated the importance of rate-limiting enzymes for product synthesis (62). Additionally, fermentation technology is an important means to improve the product synthesis. Among palmitic acid, glucose, and ethanol supplement, it was discovered that palmitic acid contributed to both cell growth and product synthesis. The addition of palmitic acid resulted in a considerable advantage in cell development after 72 h, with a rapid increase in carotenoid synthesis from 38.66 to 117.78 mg⋅L^–1^, an approximate 2-fold increase. Palmitic acid was found in most agricultural waste and was less expensive and easier to obtain than glucose, which can be repurposed as a source of carbon to produce a more valuable product.

In summary, a systematic study of the whole genome sequencing of *R. glutinis* X-20, metabolite identification, carotenoid synthesis pathway and its functional validation. The crtI and crtYB genes, obtained by genome sequencing annotation, were verified to indeed function as carotenoid synthesizers. Heterologous constructs in *S. cerevisiae* are an effective solution strategy to validate this class of organisms, and optimization of the precursor pathway and improvement of fermentation conditions facilitate further enhanced expression of gene function. This work has the potential to form the basis for studying the function of different genes in *Rhodotorula* and to guide the selection of host cells and fermentation mechanisms.

## Data Availability Statement

The datasets presented in this study can be found in online repositories. The names of the repository/repositories and accession number(s) can be found in the article/Supplementary Material.

## Author Contributions

SB designed and performed the experiments and data analysis. JG and XW contributed the bioinformatic analysis and other support. XN performed the sequencing library construction. ZL and YS supervised the writing of the manuscript throughout. GZ and JY revised the manuscript. All authors read and approved the final manuscript.

## Conflict of Interest

The authors declare that the research was conducted in the absence of any commercial or financial relationships that could be construed as a potential conflict of interest.

## Publisher’s Note

All claims expressed in this article are solely those of the authors and do not necessarily represent those of their affiliated organizations, or those of the publisher, the editors and the reviewers. Any product that may be evaluated in this article, or claim that may be made by its manufacturer, is not guaranteed or endorsed by the publisher.

## Footnotes

1
https://www.ncbi.nlm.nih.gov/cdd/
