# Supplementary material for: Carotenoid Biosynthesis: Genome-Wide Profiling, Pathway Identification in Rhodotorula glutinis X-20, and High-Level Production
Source: Front Nutr. 2022 Jun 17;9:918240. doi: 10.3389/fnut.2022.918240 (PMC9247606; doi:10.3389/fnut.2022.918240)
Supplement: Supplementary file 1 [file Data_Sheet_1.docx]

**Carotenoid biosynthesis: genome-wide profiling, pathway identification in *R. glutinis* X-20, and high-level production**

**Running title: Carotenoid biosynthesis based** **on genome** **analysis**

*Shaobo Bo, Xiaoxia Ni, Jintang Guo, Zhengyang Liu, Xiaoya Wang,* *Yue Sheng, Genlin Zhang* *^*^, Jinfeng Yang^*^*

*Key Laboratory for Green Processing of Chemical Engineering of Xinjiang Bingtuan, School of Chemistry and Chemical Engineering,* *Shihezi University, Shihezi 832000, P.R. China*

*Corresponding author

Genlin Zhang, E-mail address: zhgl_food@sina.com; Tel.: +86-993-2055015

Jinfeng Yang, E-mail address: yangjinfeng@shzu.edu.cn; Tel.: +86-993-2057159

**Table S1 Primers used in this crtI and crtYB**

| **Primers** | **Sequences** | **Recombination site** |
| --- | --- | --- |
| crtI-F | CAAGAAGCAATTAACTACATCAACTAGAACCATAATGTCTCCTC  GTCCGTCTG | Delta22 |
| crtI-R | AATGAGTGGTTAGGGACATTAAATCACTGCCTCAGGTAGACG | Delta22 |
| crtYB-F | TTGTCATATATAACCATAACCAAGTAATACATATTCAAAATGGGTG  GCTTCGACTACTG | Delta17 |
| crtYB-R | GAGTCACTTTAAAATTTGTATACACTTATTTTTTTTATAACTTCAC  AACGCCGTCCAGG | Delta17 |

The probable crtI protein has a molecular weight of 60.84 kDa and a potential isoelectric point of 7.35. Sequence research revealed that the crtI protein comprises a conserved crtI structural domain (residues 22-501), which is a characteristic of a crtI subfamily member **(Fig. S5)**. The secondary structure was utilised to predict that crtI was a mixed protein with 75.5% alpha helix, 64.6% beta pleated sheet, and 11.1% beta turn to better comprehend the molecular model of the crtI gene **(Fig. S6)**. The CDS region that encodes the crtYB sequence is 1776 bp long, comprising three ORF sections, and contains 591 amino acid sequences **(Table S3)**. The probable crtYB protein has a molecular weight of 65.39 kDa and a potential isoelectric point of 6.3. The crtYB protein comprises two CarR domains (residues 4-93), (residues 145-235), and an isoprenoid biosynthesis enzyme domain (residues 293-582) from the cl11889 super family and the Isoprenoid Biosyn C1 super family **(Fig. S7)**. This suggests that it may have a lycopene beta-cyclase function and a 15-cis-phytoene synthase function. The secondary structure was utilised to predict that crtYB was a mixed protein with 76.6% alpha helix, 45.7% beta pleated sheet, and 10.5% beta turn to better comprehend the molecular model of the crtYB gene **(Fig. S8)**.

**Table S2** **Gene sequence of crtI**

**>*Rhodotorula glutinis* X-20 crtI |OL518983|**

ATGTCTCCTCGTCCGTCTGTCATCATCGTCGGCGCAGGCGTCGGCGGCACCAGCTCGGCGGCAAGGCTCGCCCAGGCCGGCTTCGACGTCCAAGTCGTCGAGAAGAACGACTTCGCCGGCGGCCGATGCTCCCTCTTCACCGATCCGACCGGCGAGCACCGCTTCGACATGGGCCCGTCGCTCTACCTCATCCCGCGCCTGTTCGAGGAGACCTTCTCCGACCTCGGCACCAGCCTCGACGCCGAGGGCATCAAGCTCGTCAAGTGCGAGCCGAACTACCGCATCGTCTTCCCCGACAAGGAGGTCGTCGAGATGAGCAGCGACCTCACCAAGATGAAGACCGAGGTCGAGAAGTGGGAAGGCGAGAAGGGCTTCGAGGGCTTCCTCGGCTTCCTCAAGGAGGGTCACGCCCACTACGAGCTCTCGATGGTCCACGTCCTCCACCGCAACTTCACCTCGTACCTGTCGCTCCTCCGCCCGTCCTTGATCGTCAACCTCAGCAAGCTTCATCCCTTTGTGTCTGTCTACACGCGCGCGACCAAGTACTTCAAGACCGAGCGCATGCGCCGCGCCTTCACCTTTGCCGCCATGTACCTCGGCATGTCGCCGTTCGACGCTCTCGGCGCCTACAACCTCCTCCAGTACACCGAGCACTGCGAGGGCATCCTGTACCCGCTCGGCGGGTTCGGCACCGTGCCCAAGGCGCTGCAGCGCATCGCCGAGCGCAACGGCGCCAAGTTCCGTCAACTCGACCGTCAAGCGCGTCGTCGTCCAGGACGGCAAGGCGACGGGCGTCGAGCTCGAGGGCGGCGAGATTCTGTCGGCCGACATCGTCCTCGTCAACGCCGACCTCGTGTGGTCGATGGCGCACCTCTACCAGGAGACGGCCTACTCGAAGCGCCTCGAGGAGAAGCCCGTCTCGTGCTCGTCCATCTCGTTCTACTGGGCCATGAAGCGCAAGGTCCCTGAACTCGGCTCGCACACGATCTTCCTCGCCGACGAGTACAAGGAGTCGTTCGACTCGATCTTCCGCGACCACAAGATCCCGCACGAGCCGTCGTTCTACGTCAACGTTCCTAGCCGTCACGACCCCTCCGCCGCTCCCGCCGACAAGGACACGGTCATCGTCCTCGTCCCCGTCGGCCACATCTCGAAGGCGCTCCCGACGTCGTCCGACTGGGACAAGGTCGTCGAGGAGACGCGCAACAAGGTCATCCGCGAGGTCGAGGGCCGCCTCGGCATGACCAACTTCCGCGACCTCATCGCGCACGAGCAGATCAACACGCCGATCACGTGGGGCGAGAAGTTCAACCTGCACCGCGGCAGCATCCTCGGCCTCTCGCACGACTTCTTCAACGTCCTCAGCTTCCGCCCCAAGACGCGCCACCCGACCGTCAAGAACGCCTACTTCGTCGGCGCGTCGGCGCACCCGGGCACGGGCGTGCCGATCGTGCTCGCCGGTGCGCGCATGGCGACGACCCAGATCATCGACGACCTCGGCCGCCAGACGCCGCACGAGTGGAAGGTGTCGAGCAGCGAGCTCGCGACGCACCGCACGGGCCGCGACGCCCTCGGCGGCATCGGCCTCTTGATGGCGCTCGCCGCCCTCATCCTCGCCATCGCCGTCTACCTGAGGCAGTGA

**Table S3 Gene sequence of crtYB**

**>*Rhodotorula glutinis* X-20 crtYB |OL518982|**

ATGGGTGGCTTCGACTACTGGCTCGTCCATGCGCGTTGGACGATCCCTCCCTCGGTCGCGCTGTGGCTCGTGTTCCGCAAGCTGCGGACCTGGAGGGACGTGTACAAGACGCTGTTCCTCATCACGCAGATCGCTGTCACGGCGACGATACCCTGGGACTCGTACCTCATCCGGAACCGCATCTGGTCCTACCCGGACTCGTCCGTCGTCGGCCCGACCCTGTTCGCCATCCCCTACGAGGAGGTCTTCTTCTTCTTCGTCCAGACCTACCTCACCTCGACCCTGTACGCCGTCCTCACGCGCCCGATCGTCCACGCGACCCTCCTCCCGCGCACGCCGAGCGAAGGGCGCACCGTCAAGTGGACCGGCACGGCGCTGCTGTGCGGCGTCTTTGCCCTGTCGTGGGCCAAGCTCGAGGAGGGCGGCGAGGGGACCTACCTCGCCCTCATCGTCGGCTGGGTCGCGCCGTTCCTCACCTTGCTCTGGTGGGTCGCGTCTGAGCACATCTGCGCCATGCCGCGCGCGACGCTCCTCCTCGCCATCTTCGCCCCGACCGTCTTCCTGTGGGAGCTCGACGCGCGCGCTCTGCAGCGTGGCACTTGGGTCATCGAGCAGGGGACCAAGCTCGGGTGGGACTTCCGCGGGCTCGAGATCGAGGAGGCCGTCTTCTTCCTCTTGACCAACGTCATGATCGTCTTTGGCATGGCCGCCTGCGACCACTGCCTCGCCGTCCACGACCTGCGCTCGTACGACAAGGGCACCTCGTCCGTCTTCCCGCCGCTCACCGAGTTCGGCCCAATCCTCGTCAACTCGCCCGACGCCAAGCAGGGGCAGCGCATCGACGACCTGCGCGCCGCCATCGAGATCCTGTCGGTCCACTCGAAGAGCTTCTCGACGGCGAGCATGGTCTTTGACGGTCGGCTGCGGCTCGACCTCCTCTCCTTGTACGCCTGGTGCCGCGTCTGCGACGACCTCGTCGACAACGCCTCGTCGGTCGCCGCCGCCGAGGCCAACATCGACAAGATCAAGTCGTGCCTCGACCTCCTGTACCCTCGCGCGACGTCGACGCCGACCTCGCACCCCGTCGCCGTCTCGAACGACGCCATCGCCGCCGCCCTCCCCGGCTTGAGCGAGCCCGAGCGCGGCTCGTTCCGCCTCCTCGCCCTTCTCCCCATCACTCGCCCCCCCCTCGACGAGCTCCTCGCCGGCTTCCGCACCGACTTGTCGTTCCTCGCCTTCGCCGGCGAGAAGAAGAGCGCCGGCTCGAGCACGTCGATCCCCGCCGAGCTGCCGATCAAGACGGACGCCGACCTGCTCGAGTACGCCAACAACGTCGCGTCGTCGGTCGCCGACCTGTGCGTGCAGCTCGTGTGGGCGCACTGCGCCTCGTCGGTGCCCGAGCCCGAGCAGCGCGCCATCCTCGCCGCCGCGCGCGAGATGGGCCAGGCGCTCCAGCTCGTCAACATCGCGCGCGATGTGCCGGCCGACCGCGACATCCACCGCATCTACCTCCCCGGCCGGTCGCTCGACGTCGCCGTCGAGGCCATGACGCCCGACCGGCGAGAGCTCCTGCGCCGCGCGCGCGCCATGGCGGCGCACAGCCGCGAGGCGATCGAGCGCCTGCCGCGCGAGGCGAGGGGCGGGATCCGCGCGGCGTGCGACGTGTACCTGTCGATCGGCGGGGCGGTCGAGCGCGCGCTCGACGAGGGGAGGGTGCACGAGCGCGCGAGGGTCGCAAAGGGGACGAGGGCGTGGAAGGCCTGGACGGCGTTGTGA

**Table S4** **Important compounds were identified using HPLC-MS profiling in *R. glutinis* X-20**

| **Type** | **Metabolites** | **Formula** | **KEGG** | **Map name** | **Gene ID in *R. glutinis* X-20** |
| --- | --- | --- | --- | --- | --- |
| Fatty Acyls | Linolenate | C18H30O2 | map00592 | alpha-Linolenic acid metabolism | PLA2G4 (A1665) |
| Fatty Acyls | Methyl palmitate | C17H34O2 | N/A | N/A | N/A |
| Fatty Acyls | Myristic acid | C14H28O2 | map00061 | Fatty acid biosynthesis | FAS1 (A4655) |
| Fatty Acyls | 9(10)-EpOME | C18H32O3 | map00591 | Linoleic acid metabolism | PLA2G4 (A1665) |
| Prenol lipids | ATRA | C20H28O2 | map00830 | Retinol metabolism | Adhp (A5477, A6755) |
| Steroids | Estradiol-17-α | C18H24O2 | map00140 | Steroid hormone biosynthesis | KAR (A4313) |
| Steroids | Chenodeoxyglycocholate | C26H43NO5 | map04979 | Cholesterol metabolism | SOAT (A0386) |
| Other | 13-HPOT | C18H30O4 | map00592 | alpha-Linolenic acid metabolism | N/A |
| Other  Other | β-Carotene  Canthaxanthin | C40H56  C40H52O2 | map00906  map01110 | Carotenoid biosynthesis  Biosynthesis of secondary metabolites | crtI (A4526)、crtYB (A4529)  N/A |
| Other | Astaxanthin | C40H52O4 | map01110 | Biosynthesis of secondary metabolites | N/A |
| Other | L-Phenylalanine | C9H11NO2 | map01100 | Metabolic pathways | PAL (A4210) |


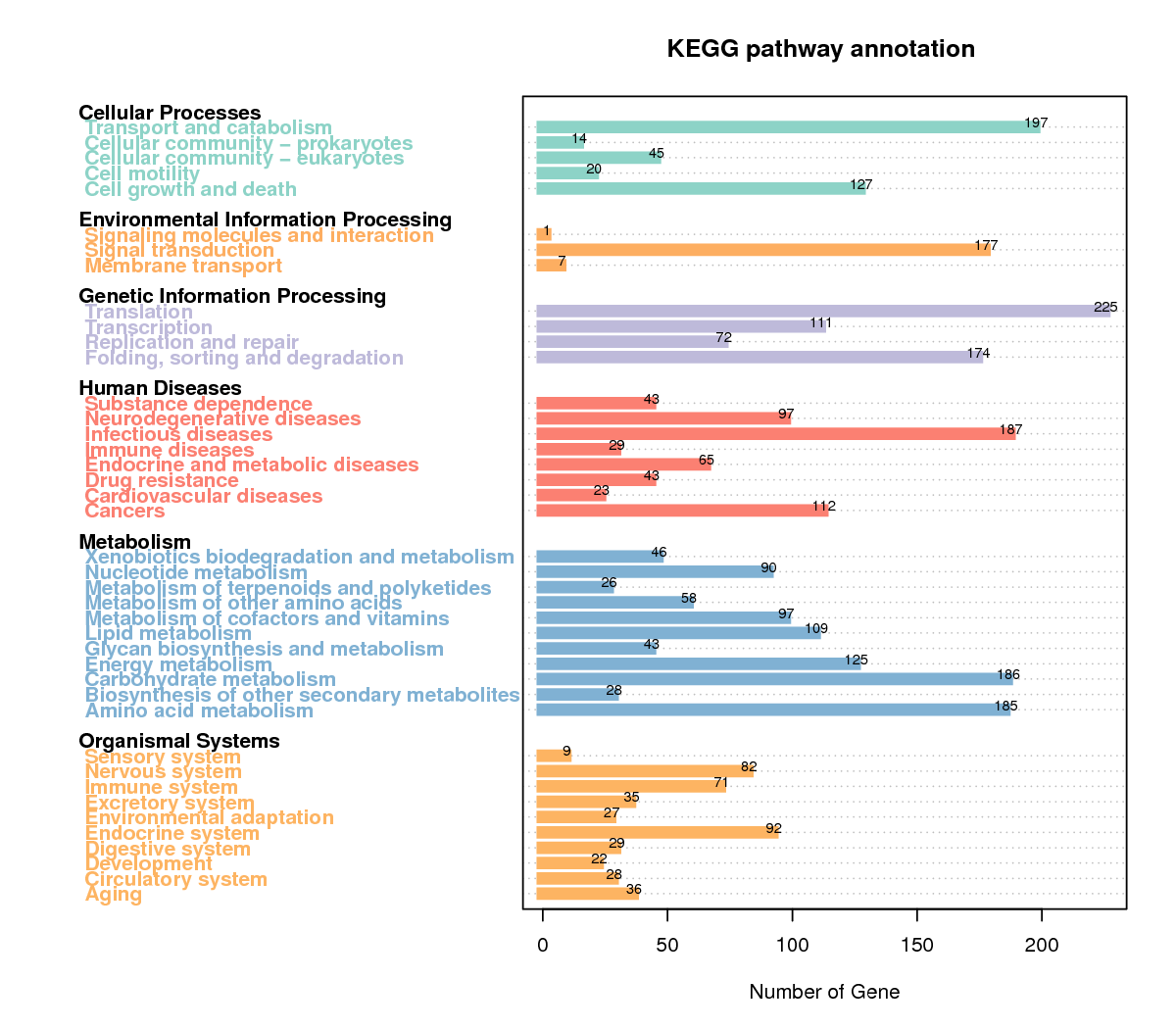


**Fig. S1** **KEGG pathway annotation:** Functional annotation of KEGG Pathways database, a total of 3584 genes were effectively mapped to 354 KEGG pathways.

As shown below, we identified the potential genes with bioindustrial applications: 1) Lipid metabolism, classified according to monounsaturated fatty acids (C18:1), saturated fatty acids (C16:0) and polyunsaturated fatty acids (C18:2), identified to include the genes encoding ACACa (acetyl CoA carboxylase), ACOX3 (acyl-CoA oxidase), PDAT (phospholipid diacylglycerol acyltransferase), PHA (phospholipase), PLA2G4 (cytosolic phospholipase A2), FAS1 (fatty acid synthase subunit beta), Adhp (alcohol dehydrogenase), KAR (17-dehydrogenase), SOAT (sterol O-acyltransferase), ADH (alcohol dehydrogenase) and HSD17B7 (estradiol dehydrogenase); 2) Fungal diseases, identified to include the genes encoding H2A (histone H2A), H3 (histone H3), H4 (histone H4), htpG (molecular chaperone HtpG), RAD51 (DNA repair protein RAD51), MSH6 (DNA mismatch repair protein MSH6), and CKS1 (cyclin-dependent kinase regulatory component CKS1); 3) Terpenoid biosynthesis, include the genes encoding GGPS1 (geranylgeranyl diphosphate synthase, type III), FDPS (farnesyl diphosphate synthase), crtI (phytonene desaturase/3,4-diehydrolycopene), crtYB (15-cis-phytoene synthase/lycopene beta-cyclase), DFT1 (farnesyl-diphosphate farnesyltransferase), ERG1 (squalene monooxygenase) and TRIT1 (tRNA dimethylallyltransferase); 4) Cellular transport and catabolism: include the genes encoding ABCB1 (ATP-binding cassette), PXA (peroxisomal long-chain fatty acid import protein), SRP54 (signal recognition particle subunit SRP54), ARF6 (ADP-ribosylation factor 6), CBL (E3 ubiquitin-protein ligase CBL); 5) Biosynthetic enzymes: including genes encoding PAL (phenylalanine ammonia-lyase), TGL2 (triacylglycerol lipase) and MGLL (acylglycerol lipase).


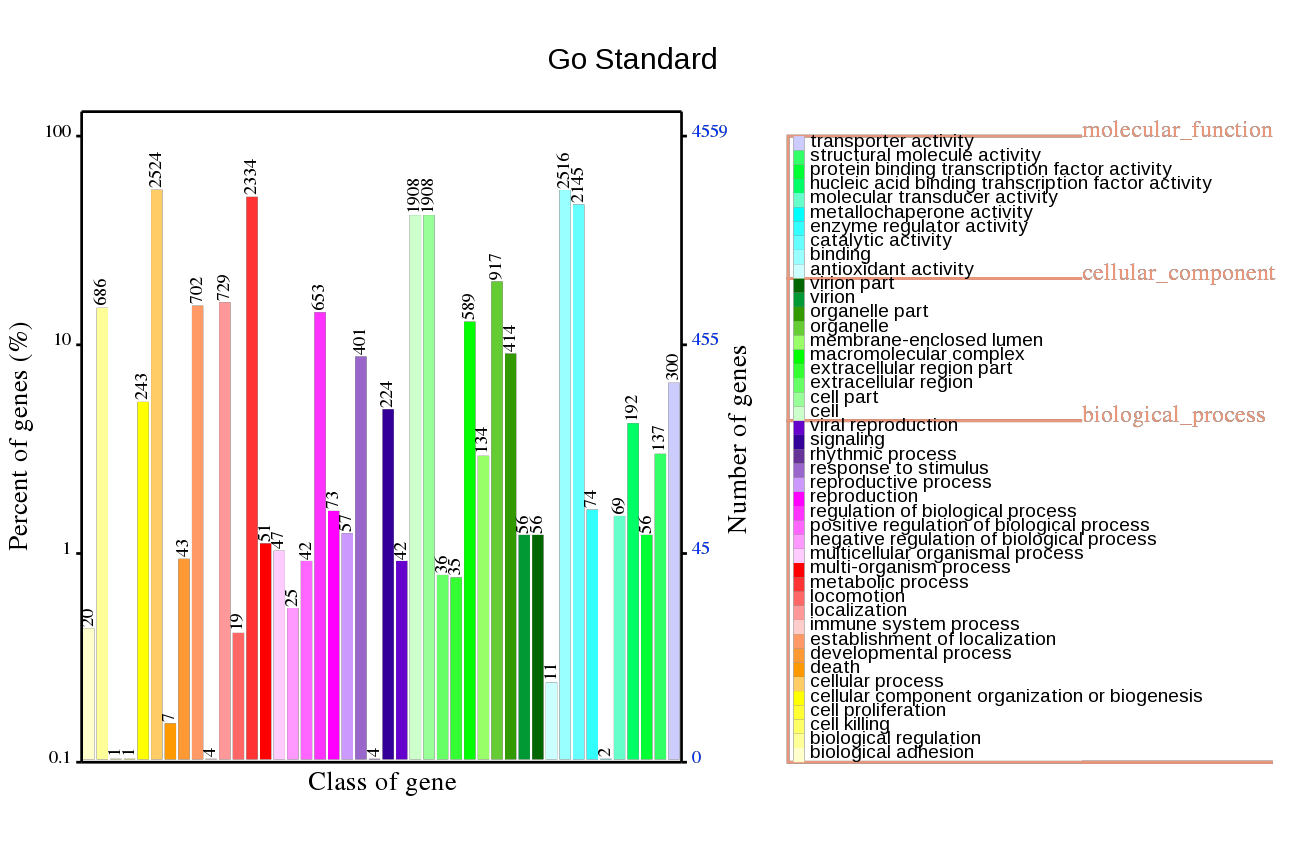


**Fig. S2** **GO pathway annotation:** There were 4,558 predicted genes could be classified into three Gene Ontology (GO) categories.


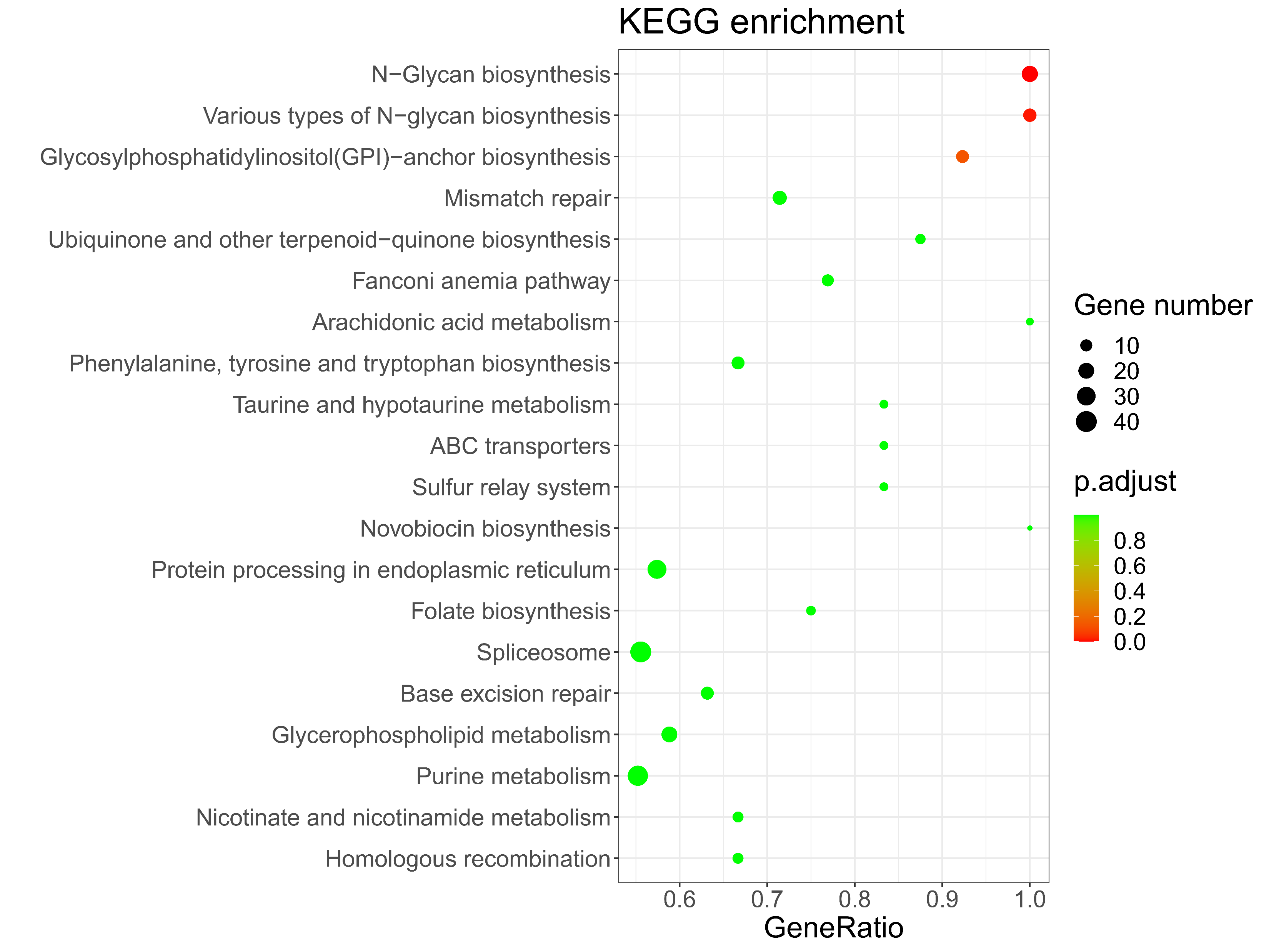


**Fig. S3** **KEGG pathway annotation of** **purifying selected genes (Ka/Ks>0.1)**

By KEGG annotation of purifying selected genes (Ka/Ks>0.1), the KEGG database enriched in orthologous genes contains “N−Glycan biosynthesis”, “Various types of N−glycan biosynthesis”, and “Glycosylphosphatidylinositol(GPI)−anchor biosynthesis”, “Mismatch repair”, “Ubiquinone and other terpenoid−quinone biosynthesis”, “Fanconi anemia pathway”, “Arachidonic acid metabolism”, “Phenylalanine, tyrosine and tryptophan biosynthesis”, “Taurine and hypotaurine metabolism”, “ABC transporters”, “Sulfur relay system”, “Novobiocin biosynthesis”, “Protein processing in endoplasmic reticulum”, “Folate biosynthesis”, “Spliceosome”, “Base excision repair”, “Glycerophospholipid metabolism”, “Purine metabolism”, “Nicotinate and nicotinamide metabolism”, “Homologous recombination”.


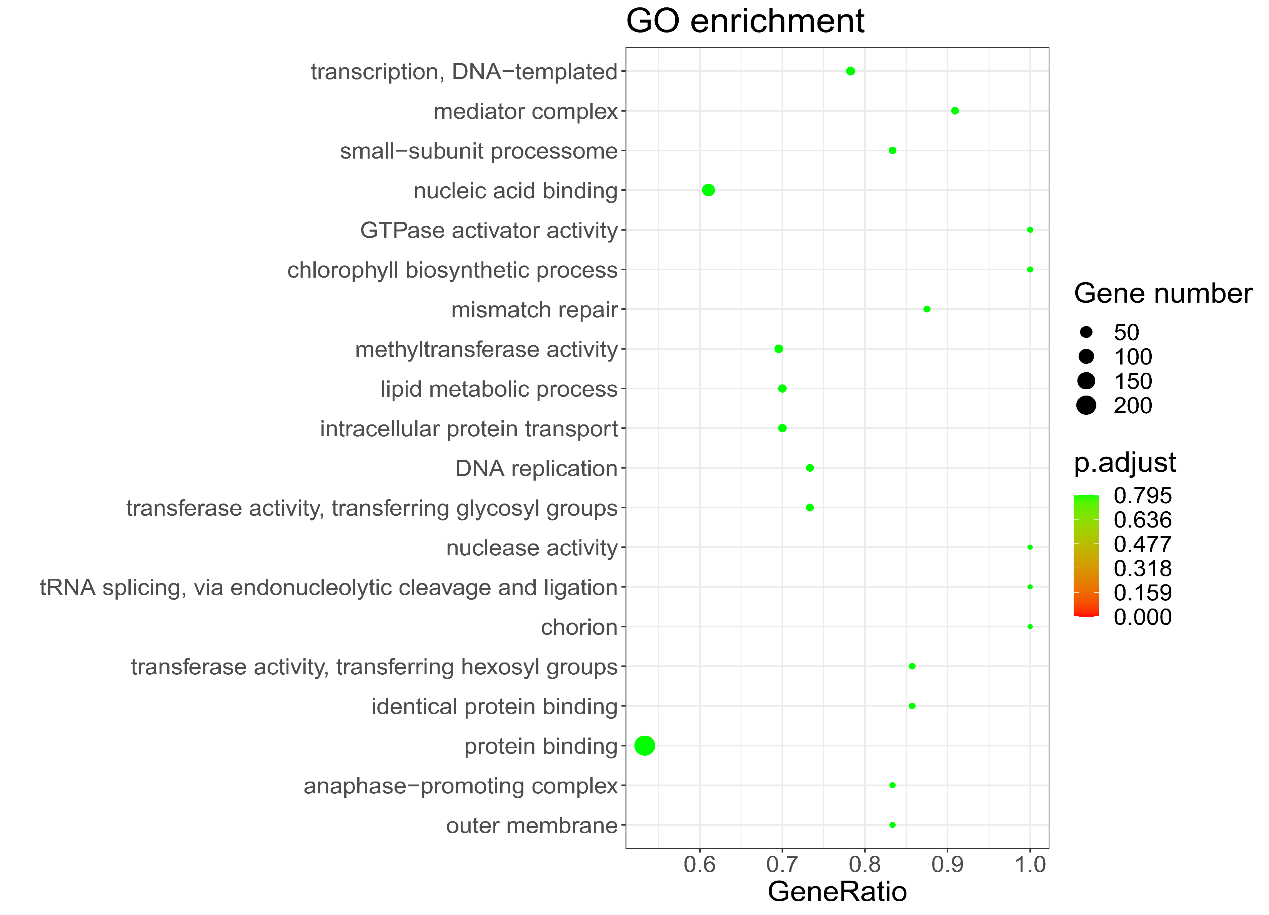


**Fig. S4** **GO pathway annotation of** **purifying selected genes (Ka/Ks>0.1)**

They were processed to GO annotation, and the GO class enriched classes in the orthologous genes were included “transcription, DNA−templated”, “mediator complex”, “small−subunit processome”, “nucleic acid binding”, “GTPase activator activity”, “chlorophyll biosynthetic process”, “mismatch repair”, “methyltransferase activity”, “lipid metabolic process”, “intracellular protein transport”, “DNA replication”, “transferase activity, transferring glycosyl groups”, “nuclease activity”, “tRNA splicing, via endonucleolytic cleavage and ligation”, “chorion”, “transferase activity, transferring hexosyl groups”, “identical protein binding”, “protein binding”, “anaphase−promoting complex”, “outer membrane”.


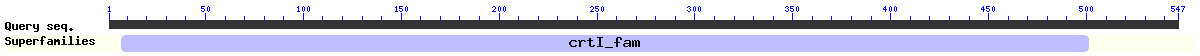


**Fig. S5 The CDS region encoding the crtI sequence**


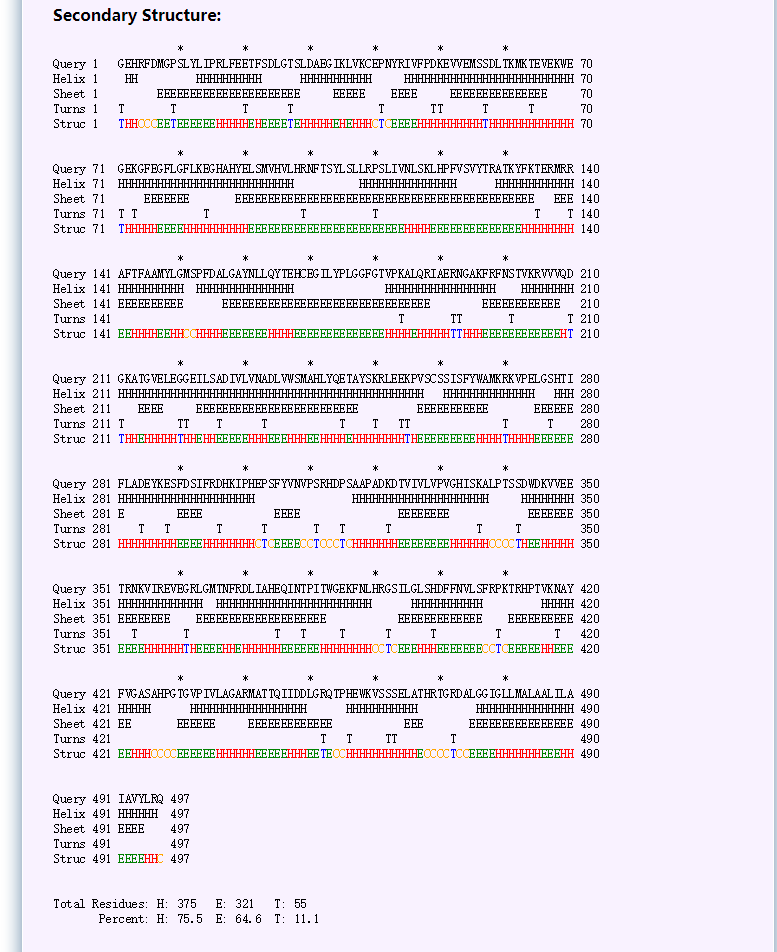


**Fig. S6 The secondary structure of crtI**


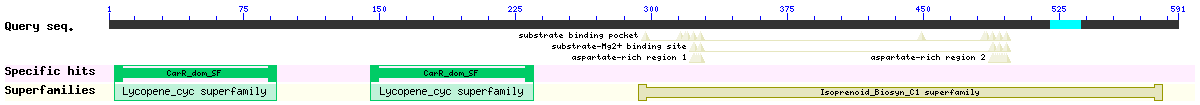


**Fig. S7 The CDS region encoding the crtYB sequence**


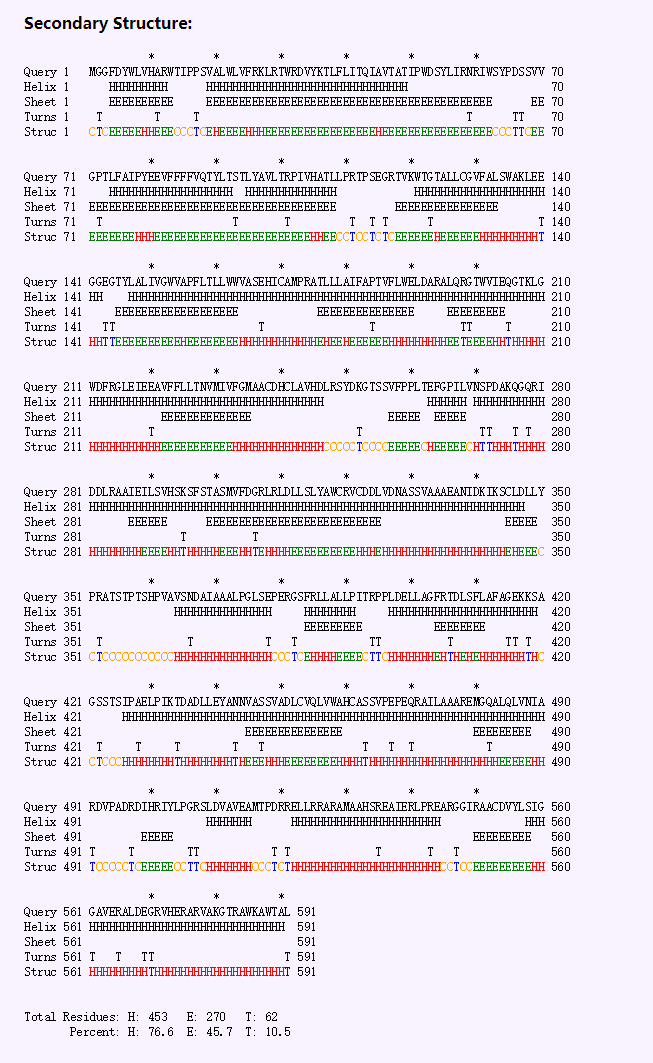


**Fig. S8 The secondary structure of crtYB**

**Fig. S7** **Fermentation product testing information.** B1: *Sc*. CEN (B1); B2: *Sc.* INV (B2); B3: *Sc*.CEN (B3); B4: *Sc*. INV (B4).
